# Total reflection X-ray fluorescence spectrometry for trace determination of iron and some additional elements in biological samples

**DOI:** 10.1007/s00216-020-02614-8

**Published:** 2020-04-26

**Authors:** Andreas Gruber, Riccarda Müller, Alessa Wagner, Silvia Colucci, Maja Vujić Spasić, Kerstin Leopold

**Affiliations:** 1 Institute of Analytical and Bioanalytical Chemistry, Ulm University, 89081 Ulm, Germany; 2 Institute of Comparative Molecular Endocrinology, Ulm University, 89081 Ulm, Germany; 3 Department of Pediatric Hematology, Oncology and Immunology, University of Heidelberg, 69120 Heidelberg, Germany; 4 Molecular Medicine Partnership Unit, 69120 Heidelberg, Germany

**Keywords:** Total reflection X-ray fluorescence spectrometry, Iron trace analysis, Biometal trace analysis, Bone marrow–derived macrophages, Liver cells, Liver tissues

## Abstract

Trace elements are essential for life and their concentration in cells and tissues must be tightly maintained and controlled to avoid pathological conditions. Established methods to measure the concentration of trace elements in biological matrices often provide only single element information, are time-consuming, and require special sample preparation. Therefore, the development of straightforward and rapid analytical methods for enhanced, multi-trace element determination in biological samples is an important and raising field of trace element analysis. Herein, we report on the development and validation of a reliable method based on total reflection X-ray fluorescence (TXRF) analysis to precisely quantify iron and other trace metals in a variety of biological samples, such as the liver, parenchymal and non-parenchymal liver cells, and bone marrow–derived macrophages. We show that TXRF allows fast and simple one-point calibration by addition of an internal standard and has the potential of multi-element analysis in minute sample amounts. The method was validated for iron by recovery experiments in homogenates in a wide concentration range from 1 to 1600 μg/L applying well-established graphite furnace atomic absorption spectrometry (GFAAS) as a reference method. The recovery rate of 99.93 ± 0.14% reveals the absence of systematic errors. Furthermore, the standard reference material “bovine liver” (SRM 1577c, NIST) was investigated in order to validate the method for further biometals. Quantitative recoveries (92–106%) of copper, iron, zinc, and manganese prove the suitability of the developed method. The limits of detection for the minute sample amounts are in the low picogram range.

**Electronic supplementary material:**

The online version of this article (10.1007/s00216-020-02614-8) contains supplementary material, which is available to authorized users.

**Figure:**
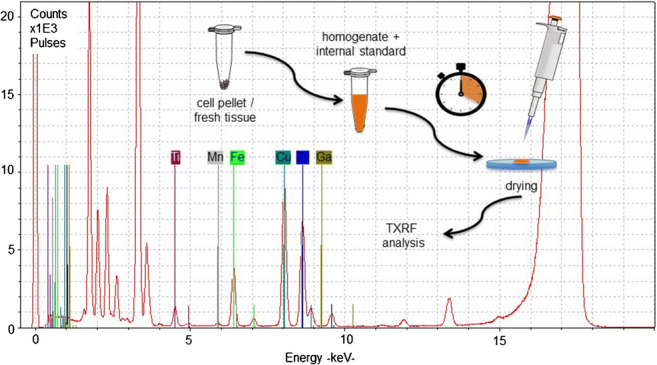
Graphical abstract

## Introduction

Essential trace elements, such as iron, zinc, copper, manganese, and selenium, are required to maintain physiological homeostasis in almost all organisms, as they are contained within proteins which are involved in numerous cellular and systemic processes, including oxygen transport, cellular respiration, regulation of transcription and DNA repair, immune defense, and metabolism [1]. A deficiency as well as an excess of these elements can cause and/or indicate various diseases [2, 3]. The provision of suitable analytical methods for trace elements’ determination in biological samples is therefore important for diagnosis and treatment of diseases as well as for medical research. For the latter, often tissue and/or cell samples are investigated in order to understand molecular and biochemical processes and inter-relationships. A widely used and established approach for iron determination in this field involves staining of the samples and photometric detection, i.e., colorimetric assays using most commonly ferrozine as complexing and staining agent [4–6]. Besides, instrumental atomic spectrometric techniques for quantification, such as graphite furnace atomic absorption (GFAAS), have been used [7–9]. This technique can also be applied to suspensions, slurries, and even solids; however, it is restricted to the determination of single elements and requires external calibration. Alternatively, inductively coupled plasma-mass spectrometry (ICP-MS) or inductively coupled plasma-optical emission spectrometry (ICP-OES) was suggested [10–17]. These techniques provide multi-element determination; however, complete dissolution of the samples is required, involving therefore time- and reagent-consuming sample preparation [18].

In recent times, total reflection X-ray fluorescence spectrometry (TXRF) is increasingly proposed in trace element determination in biological samples as it combines multi-element determination with the possibility of applying suspensions and/or solids [19–21]. Furthermore, TXRF is cost-effective and does not require any gas or cooling media. Examples for valid trace determination by TXRF cover a broad variety of different biological samples, from isolated proteins and nuclei acids to fluids, cells, bacteria, hair, and tissues such as the liver, placenta, chest, kidney, lung, prostate, or colon [2, 13, 20–33]. Besides the above-mentioned advantages, highly convenient and reliable multi-element quantification can be performed by adding an internal standard for a one-point calibration [20, 21, 27, 30, 31]. In addition, trace element determination by TXRF requires only minute sample amounts providing detection limits in the picogram range [19, 30]. This issue is beneficial with regard to biomedical research, since here the obtained sample amounts are often limited and a pooling of individual samples has to be avoided in order to maintain the information on biological variability of trace elements in the samples.

In this work, biomedical samples originate from mouse model studies, with each sample coming from an individual mouse. Hence, sample weights are in the low milligram range for liver tissues and in the low to sub-microgram range for cells. Moreover, isolation of different cell types from the liver (i.e., parenchymal and non-parenchymal liver cells) is a very sophisticated and elaborative procedure and the isolated cells cannot be cultivated since this might deteriorate the trace element levels. The general biomedical scope of these studies is the investigation of the iron metabolism since the excess and the deficiency of iron contribute to global health burden [1]. Hence, the determination of iron content in the liver, parenchymal and non-parenchymal liver cells, and bone marrow–derived macrophages is in the focus [34]. Therefore, the aim of this work was to develop and validate an analytical method for iron quantification and determination of further trace elements in minute biological samples using TXRF. The method is characterized by high sensitivity, accuracy, and precision providing at the same time straightforward sample preparation, high sample throughput, and simple procedure.

## Materials and methods

### Reagents and materials

Ultrapure water (UPW) obtained from ultrapure water purification system (Sartorious AG, Göttingen, Germany) was used for all operations. Nitric acid (HNO_3_, 63% AnalR Normapur, VWR International GmbH, Darmstadt, Germany) was pre-cleaned by subboiling distillation (dst1000, Salvillex Coporation, USA) and used for digestion of samples, acidification of standards, and cleaning procedures.

All containers and materials (pipette tips, cups, etc.) were cleaned by standard trace metal procedures including a two-step leaching procedure for at least 24 h in 10% nitric acid baths at room temperature. After rinsing, materials were stored in 0.5% nitric acid until usage. Prior to usage, they were rinsed three times with UPW.

Gallium and titanium standards for TXRF quantification were prepared by adequate dilution of 1000 mg/L Ga (in 2% HNO_3_, VWR International GmbH, Darmstadt, Germany) or 1000 mg/L Ti (in 2% HNO_3_, Sigma Aldrich, Darmstadt, Germany), respectively. Iron standard solutions for external calibration of GFAAS were obtained from appropriate dilution of 1000 mg/L Fe (in 0.5 mol/L HNO_3_ Merck KGaA, Darmstadt, Germany) in 63% HNO_3_.

Quartz glass TXRF sample carriers (diameter 30 mm, height 3 mm, Bruker Nano GmbH, Berlin, Germany) were rinsed successively with UPW and acetone (technical, VWR International GmbH, Darmstadt, Germany). Then, carriers were placed first in basic cleaning solution (Hellmanex III, Hellma Analytics GmbH & Co KG, Mühlheim Germany) at 80 °C for at least 1.5 h and second, after three times rinsing with UPW, in 10% nitric acid for another 1.5 h. After rinsing the carriers again three times with UPW, they were placed in a drying chamber at 120 °C for 2 h. After cooling down to room temperature, the carriers were siliconated in the center with 10 μL of silicone solution (SERVA electrophoresis GmbH, Heidelberg, Germany).

Efficiency of purification and cleaning procedures was regularly checked by blank measurements of all used reagents and materials.

### Animal experimentations (liver tissues)

Wild-type mice were maintained on a standard mouse diet containing 200 mg/kg iron (Ssniff, Soest, Germany) under a constant dark-light cycle and were allowed access to food and water ad libitum. All animal experiments were approved by and conducted in compliance with the guidelines of the University Ulm Animal Care Committee. Mice were sacrificed by CO_2_ inhalation and the livers were collected and kept at − 80 °C.

### Liver cells’ separation

Liver parenchymal cells (hepatocytes, HCs), and non-parenchymal cells such as resident macrophages Kupffer cells (KC), liver sinusoidal endothelial cells (LSECs), and hepatic stellate cells (HSC), were isolated as previously described [35]. Following isolations, cells were spin-down (or centrifuged) and the cell pellets were kept at − 80 °C until further analysis.

### Preparation of bone marrow–derived macrophages

Bone marrow–derived macrophages (BMDMs) were prepared as previously described [34]. Briefly, marrows from femur bones of wild-type mice were flushed and cells were plated at density 1 × 10^6^ cells/mL in culture petri dishes (Becton Dickinson, USA) using Dulbecco’s minimal Eagle’s medium (DMEM; Invitrogen, USA) supplemented with 10% fetal bovine serum, 10 mM sodium pyruvate, 10 mM l-glutamine, penicillin, and streptomycin (Sigma Aldrich, St. Louis, USA). The bone marrow cells were differentiated into macrophages using DMEM media supplemented with 20% mouse L929 fibroblast cell line culture (as available at the institute [36]) supernatant as a source for macrophage colony-stimulating factor. Following 4 days of culture, non-adherent cells were removed and adherent cells, bone marrow–derived macrophages (BMDMs) were washed twice with PBS, and the medium was replaced daily for a week. At the end of the culturing, BMDMs were collected and stored at − 80 °C.

### Sample preparation for trace metal measurements

#### Bovine liver (SRM 1577c)

The standard reference material SRM 1577c “bovine liver,” available from the National Institute of Standards and Technology (NIST), was used for validation of Fe, Cu, Zn, and Mn determination by TXRF. For this purpose, 100 mg of the bovine liver sample was suspended in 4 mL subboiled nitric acid. Homogenization, i.e., partial digestion, was achieved by alternating vortexing (4× 60 s, 2500 U min^−1^) and ultrasonication (3× 5 min, 40 °C). Finally, 500 μL of this suspension was mixed vigorously with 500 μL subboiled HNO_3_ and 10 μL of Ti standard solution. An aliquot of this homogenate is then applied to a sample carrier and measurement by TXRF is performed as described in the “General measurement procedures” section. Agreement of measured to certified values was evaluated by calculation and comparison of measurement differences and combined, extended uncertainties, as described by [37].

#### Liver cells and BMDMs

Prior to measurement liver cells and BMDMs were defrosted. Subsequently, 1 mL of HNO_3_ was added for (partly) digestion and the samples were vigorously mixed for 15 s at 2500 rpm. Then, 10 μL of a Ga standard solution (100 mg/L in 2% HNO_3_) was added as internal standard and for TXRF quantification homogenized for another 60 s at 2500 rpm. An aliquot of the respective suspension is then applied to a TXRF sample carrier or GFAAS sample vial. In some cases, for GFAAS measurement, appropriate dilution in HNO_3_ was performed in order to meet the working range.

#### Liver tissues

Prior to measurement, liver tissues were defrosted and sample weight was recorded. Then, 1 mL HNO_3_ was added and the samples were vigorously mixed for 60 s at 2500 rpm for (partly) digestion. Subsequently, the suspensions were treated in an ultrasonic bath for 15 min at 40 °C. Afterwards another mixing step of 60 s and 2500 rpm was performed. Finally, 10 μL of Ti standard solution (100 mg/L in 2% HNO_3_) was added as internal standard for TXRF quantification, and the suspension was mixed for another 60 s at 2500 rpm. An aliquot of the respective suspension is then applied to a TXRF sample carrier. For GFAAS measurement, samples were diluted by HNO_3_ with appropriate factors.

All details on preparation of biological samples are summarized in the Electronic Supplementary Material (ESM) Table S1.

#### General measurement procedures

For TXRF measurements, 10 μL of a homogenate was applied to the center of a quartz glass sample carrier and placed on a heating plate (60 °C) until complete dryness. To minimize contaminations, the plate was covered with a glass dish and the procedure was performed in a class 100 laminar flow box (Susi Super Silent, Spetec GmbH, Erding, Germany).

Recovery experiments using GFAAS as a reference method were performed for validation of Fe determination by TXRF in all samples. Calibration of GFAAS was performed using six concentrations and 3 replicate measurements. Calibration ranges and further details are given in ESM Table S1. In order to fit into the limited linear range of AAS calibration, several samples were adequately diluted (see ESM Table S1).

#### Instrumentation

TXRF measurements were performed using a high-efficiency module S2 Picofox (Bruker Nano GmbH, Berlin, Germany) equipped with Mo X-ray tube. Excitation of the sample was carried out using a voltage of 50 kV and a current of 600 μA. Measurement live time was set to 1000 s or 500 s, respectively. Evaluation of the obtained spectra was achieved using Spectra PicoFox (7.2.5.0, Bruker Nano GmbH) software.

GFAAS measurements of iron were carried out using a novAA800 (Analytik Jena AG, Jena, Germany) equipped with an AS-GF auto sampler for liquid sampling (Analytik Jena AG). For excitation, a Fe hollow cathode lamp (Analytik Jena AG) was applied using the wavelength of 248.3 nm and a slit width of 0.5 nm. The background was corrected by usage of a deuterium hollow cathode. Argon with a purity of 99.996% (MTI, Neu-Ulm, Germany) was used as purge and protective gas. The applied sample volume was set to 20 μL and the furnace temperature program is given in ESM Table S2. For each sample, three replicates were measured and the mean value was used for further calculations.

All procedural steps critical to contamination were carried out in a class 100 laminar flow box (Susi Super Silent).

#### TXRF spectrum evaluation and statistics

To TXRF spectra, a profile Bayesian deconvolution was applied (normal fit, max. stripping cycles: 40). All calculations were performed by S2 PICOFOX Control software.

The lower limit of detection (LLD) of the element *i* was calculated using the following equation:

$$ \mathrm{L}{\mathrm{LD}}_i=\frac{3\ {c}_i\sqrt{N_{\mathrm{BG}}}}{N_i} $$

with *c*_*i*_, concentration of the element *i*; *N*_*i*_, area of the fluorescence peak in counts; and *N*_BG_, background area subjacent the fluorescence peak.

Quantitative evaluation was achieved using an internal standard (IS) and correlating its concentration with respect to the relative sensitivities to the net intensities found for the analyte and the IS as given by the following equation:

$$ {c}_{\mathrm{A}}=\frac{\frac{N_{\mathrm{A}}}{S_{\mathrm{A}}}}{\frac{N_{\mathrm{IS}}}{S_{\mathrm{IS}}}}\bullet {c}_{\mathrm{IS}} $$

with *c*_A/IS_, concentration of the analyte/internal standard; *N*_A/IS_, net intensity of the analyte; and *S*_A/IS_, relative sensitivity of the analyte/internal standard. Net intensity of the elements can be calculated from theory and is independent from matrices.

Reproducibility of TXRF measurements was evaluated by replicate measurement of sample carriers and preparation of individual sample carriers. Each carrier was measured 4 times by TXRF, rotating the sample carrier 90° in-between measurements (*n*_R_ = 4: 0°, 90°, 180°, 270°). In addition, at least 2 individual sample carriers were prepared with aliquots of each digest. If the measured Fe concentration differed by more than 10% between the individual carriers, 3 additional carriers were prepared (*n*_C_ = 2–6). Statistic evaluation of these data sets was performed using the robust parameter of value range, because other parameters like variance or standard deviation are unsuitable for *n* ≤ 6. Ranges were calculated as the difference between the observed minimum and maximum values for each sample. Finally, the median was calculated for each sample series, i.e., biological sample type with *N* individual samples. *N* was between 5 and 12. The resulting median ranges are expressed in micrograms per liter and additionally converted into percent values dividing by median concentration of the corresponding sample type.

## Results and discussion

**Fig. 1:**
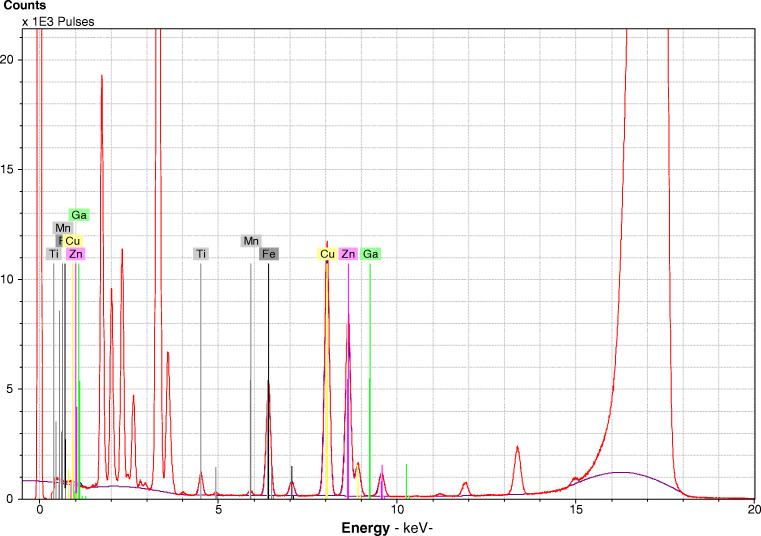
Exemplary TXRF spectrum of standard reference material *bovine liver* (SRM 1577c from NIST)

The TXRF method development was performed by optimization of sample preparation and measurement parameters, namely homogenization, selection of internal standard, and measurement time, as well as determination of analytical figures of merits. Finally, comprehensive examination of validity of the suggested method for all sample types was performe. Thereby, measurement of the matrix reference material bovine liver SRM 1577c as well as measurement of all real samples using GFAAS as a reference method was accomplished. An exemplary TXRF spectrum is presented in Fig. 1.

### Optimization of sample preparation

Sample preparation is a critical point in trace element analysis. This is true also in the context of biological samples, where appropriate sample digestion is crucial to obtain valid data [12]. Incomplete sample digestion can lead to falsified results and/or high variances. However, the impact of incomplete digestion of biological matrices depends strongly on the measurement technique that follows. While residual solids in a digest affect sample inlets with spray disperser, this is not necessarily the case in TXRF. Here, slurries and suspension can be applied onto the sample carrier and a quantitative measurement is possible, when the following conditions are fulfilled: (A) The analyte is homogenously dispersed in the suspension; (B) Addition of internal standard results in a homogenous distribution; (C) Application and drying of the suspension on the reflector (sample carrier) are reproducible.

The feasibility of such approach has recently been confirmed by Espinoza-Quiñones et al. [38] who investigated several matrix reference materials applying solid suspensions prepared by using pure water. Agreeable precision and accuracy, as well as sufficient limits of detection for trace and major elements, were obtained by TXRF analysis, whereas Marguí et al. [21] found minor recoveries in comparison with ICP-OES analysis of digests when suspending dried and grinded human placenta in surfactant-containing water for TXRF analysis. Anyway, the studied reference materials and the placenta samples were ideally homogenized and dried materials, whereas the fresh, real samples investigated in this work (sections of mouse liver and cell samples) were not pre-homogenized, as any manipulation of the minute sample amounts was avoided. Instead, the oxidation power of nitric acid for partly decomposition of the matrix was used. Hence, the straightforward sample preparation proposed here consists of a digestion procedure using concentrated nitric acid at room temperature and stirring and/or sonication for a few minutes. Thereby, cells are lysed and the organic material of the tissues is partly decomposed; however, no complete digestion is achieved. In order to prove the suitability of this procedure, reproducibility of measurement results was checked first. Thereby, replicate measurement of an individual sample carrier was evaluated in order to confirm the homogeneity of the applied digest on the carrier. For this purpose, each carrier was measured 4 times by TXRF, rotating the sample carrier 90° in-between replicate measurements. Moreover, reproducibility in sample carrier preparation and homogeneity of the digest itself were also studied. Therefore, 2 to 6 aliquots of each homogenate were applied to individual sample carriers. The observed median ranges resulting from rotation of sample carriers or from individual sample carriers are presented in Table 1.

**Table 1:** Reproducibility of TXRF analysis obtained from replicate measurement of Fe in sample digests given as median concentration range

Sample type		Number of biologically individual samples	Median concentration in μg/L	Median ranges observed as a result of ...			
				(a) Sample carrier rotation (*n*_R_ = 4)		(b) Multiple sample carrier preparation (*n*_C_ = 2 to *n*_C_ = 6)	
				μg/L	%	μg/L	%
Liver cells	KC	*N* = 12	66.80	3.38	5.06	1.68	2.51
	HC	*N* = 9	1100.26	13.76	1.25	20.56	1.87
	LSEC	*N* = 12	71.13	4.19	5.89	3.52	4.95
	HSC	*N* = 5	17.34	7.71	44.43	4.04	23.27
Macrophages BMDM		*N* = 12	128.60	3.81	2.97	3.89	3.03
Liver tissues		*N* = 8	421.56	9.09	2.16	13.52	3.21

The different biological sample types cover Fe contents in the homogenates from the low to high micrograms per liter range, as can be seen in Table 1. With the exception of hepatic stellate cells (HSC) of the liver, observed median ranges in percent of median Fe concentration are between 1.25% and maximum 5.89%, which is perfectly acceptable for trace metal analysis. HSC samples showed generally low iron levels and accordingly the percentage values for the found ranges are higher. Anyway, the values between 3.38 and 13.76 μg/L for replicate measurement of an individual sample carrier are comparable with those for multiple carrier preparation (1.68 to 20.56 μg/L). These data clearly confirm the reproducibility of the suggested procedure, i.e., the proposed digestion achieves sufficient homogeneity and application of a few microliters on the sample carriers followed by drying is reproducible.

In these preliminary studies, also the feasibility of application of different internal standards was tested. Here, the absence of the selected element in the original samples and the absence of overlapping energy bands in the spectrum with analytes are prerequisites. X-ray fluorescence spectra of sample homogenates without addition of any standards revealed that gallium is not present in any of the samples. However, a potential overlap with energy lines of gallium with zinc was observed in the case of the liver tissues, where relatively high Zn contents were found. Here, Kα lines of Ga (Kα_1_ = 9.2506 keV; Kα_2_ = 9.2238 keV) may be interfered by high Zn signals (Kα_1_ = 8.6372 keV; Kα_2_ = 8.6141 keV; Kβ_1_ = 9.5704 keV). Hence, for liver samples, titanium (Ti) is a better option, which originally was absent in these samples, whereas in the BMDMs, Ti was observed. Therefore, cell samples were investigated using Ga as internal standard, whereas for liver tissues, Ti was used.

**Fig. 2:**
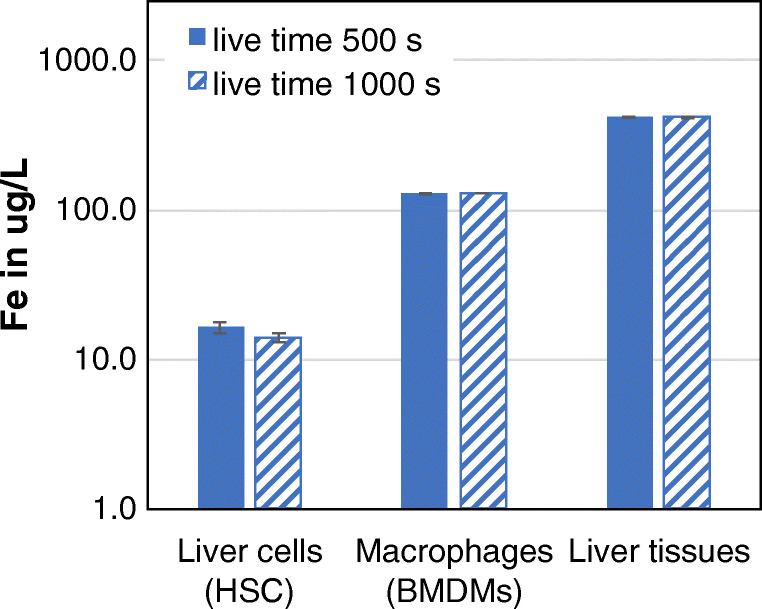
Comparison of results applying different live times for TXRF measurement for exemplary sample types (median values with *N* = 5 (HSC); *N* = 12 (BMDMs); *N* = 8 (liver tissues) and *n* ≥ 2; error bars represent median measurement error as calculated from the spectra)

Finally, measurement time, more precisely acquire or live times of TXRF measurement, was varied. The results for 500 s vs. 1000 s live time for 3 exemplary sample series are given in Fig. 2 and show no significant differences regardless the concentration range. Therefore, all further measurements were performed with a live time of 500 s.

### Method validation

**Fig. 3:**
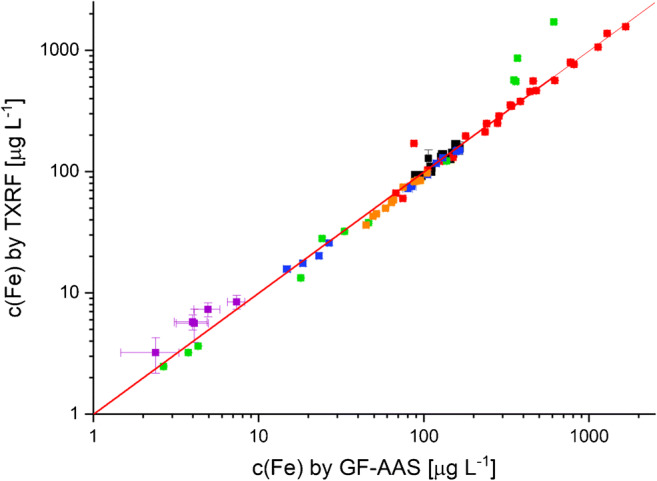
Recovery function for validation of Fe determination in liver cells (, HSC; , HC; , LSEC; , KC), macrophages (, BMDM), and liver tissues () by TXRF using GFAAS as a standard reference method (recovery function: *y* = (0.9993 ± 0.0014)*x* − (2.8456 ± 0.3332); error bars represent uncertainties as derived from calibration prognosis interval for GFAAS measurement and from errors calculated from the spectra for TXRF measurements)

Accuracy and precision of iron determination using the above-described procedure were assessed in a series of recovery experiments using GFAAS as a reference method for all real samples. The results are presented in Fig. 3.

A large concentration range from approximately 1 to 1600 μg/L Fe in the homogenates is covered by the investigation of the different sample types. The recovery function includes 77 individual samples and reveals a very good accuracy with a recovery rate of 99.93 ± 0.14% and an intercept of nearly zero. Therefore, any systematic errors using the proposed TXRF method for Fe trace analysis in the studied biomedical sample matrices can be excluded. In addition, a high precision was obtained which is comparable with GFAAS measurement.

Further validation was achieved by investigation of the standard reference material *bovine liver* (SRM 1577c from NIST). Here, recoveries of the certified trace elements in the microgram per gram range were investigated. In Table 2, the certified and found concentrations are compared.

**Table 2:** Recoveries of trace elements in SRM 1577c bovine liver. *n.d.a.*, no data available; *n.d.*, not detected

Element	Certified value (μg/g)	Found value (μg/g)	Recovery (%)
Ca	131 ± 10	n.d.a.^b^	-
Co	0.300 ± 0.018	n.d.a.^c^	-
Cu	275.2 ± 4.6	291.7 ± 22.0	106 ± 8
Fe	197.94 ± 0.65	195.96 ± 19.79	99 ± 10
Mg	620 ± 42	n.d.	-
Mn	10.46 ± 0.47	9.62 ± 1.15	92 ± 11
Mo	3.30 ± 0.13	n.d.a.^d^	-
Se	2.031 ± 0.045	1.584 ± 0.183^e^	78 ± 9
Zn	181.1 ± 1.0	188.3 ± 21.7	104 ± 12
Rb	(35.3 ± 1.1)^a^	30.4 ± 3.5^e^	86 ± 10

^a^Indicative value, not certified; ^b^peaks are overlapping with energy lines of potassium; ^c^peaks are overlapping with Kβ lines of iron; ^d^no quantification possible due to usage of Mo X-ray source; ^e^significant difference between reference and found values

Accurate and precise determination of the trace metals Cu, Fe, Mn, and Zn is simultaneously possible using the proposed TXRF method. The elements Se and Rb were also determined, but show minor recoveries. In the case of Se, concentration in the material is very low and close to the detection limit of the TXRF method. Digestion of the sample in a smaller reagent volume, however, may allow accurate Se determination. Rb value given in the certificate is only indicative and further investigations by other methods could clarify if Rb quantification by the suggested TXRF method is possible.

### Analytical figures of merit and comparison with other methods

In addition to accuracy and precision, further analytical figures of merit of the newly developed TXRF method were determined. Table 3 presents the found values and clearly confirms the suitability of the proposed TXRF method for trace element determination in minute biological samples.

**Table 3:** Analytical figures of merit

Applicable sample weights	1.6 μg–27.6 mg	
Applied digest volume	10 μL	
Sample preparation time^a^	1 min^b^/20 min^c^	
Measurement time (live + dead time)	< 12 min	
Validated working range (Fe)	0.3–1600 μg/L	
Lower limits of detection^d^:		
Element	in μg/L	in pg
Cu	0.46–0.78	4.6
Fe	0.32–1.53	3.2
Mn	1.06–1.71	10.6
Se	0.32–0.49	3.2
Zn	0.44–0.89	4.4
Rb	0.38–0.59	3.8

^a^Including digestion, homogenization, and mixture with internal standard for ^b^cells, and ^c^tissues; ^d^lower limits of detection were calculated from the spectra as detailed in the experimental part

Furthermore, the performance of the proposed method was compared with that of other methods reported recently in literature (see Table 4). Most common practices for iron determination in the field of biomedical research have been and still are colorimetric assays, i.e., photometric detection after adequate staining of the samples (e.g., [4, 5]), and/or application of GFAAS (e.g., [5, 7, 9]). Sensitivity of colorimetric assays is typically much lower than that of atomic spectrometric techniques, allowing detection of Fe in a concentration range between a few micrograms per liter to low milligrams per liter. GFAAS may achieve slightly lower detection limits (in this work LOD = 0.4 μg/L), but its linear range is quite restricted, which requires additional sample dilution when higher Fe concentrations are present [7]. More importantly, both methods provide only single element information. In order to overcome these limitations, lately investigations on the applicability of modern multi-element atomic spectrometric techniques, like ICP-OES and ICP-MS and most recently TXRF, are in focus. As stated above, for ICP-based techniques, total decomposition of the biological sample matrix, i.e., introduction of a clear solution, is a prerequisite for reproducible and valid trace quantification. Therefore, sample pre-treatment yields at total digestion and is often performed using aggressive acid mixtures for several hours at elevated temperature and/or by microwave assistance [10–12, 14, 15]. Furthermore, the investigated sample amounts are, due to practical reasons of sample preparation, rather high in comparison with the minute sample amounts applied in this work. A fewer number of publications deal with the investigation of iron and other trace metals in tissues or cells using TXRF. Varga et al. [13], for example, compare ICP-MS techniques with TXRF for the determination of trace metals in human liver, and as can be seen from the achieved recoveries (Table 4), analytical performance is comparable. Wrobel et al. [24] combine TXRF with μXRF for quantitative Fe, Cu, and Zn imaging in rat kidney, spleen, and liver tissues. Matusiak et al. [23] apply TXRF for Ca, Fe, Cu, and Zn quantification in rat liver tissues. However, for the latter, no validation experiments are given. In contrast, Marguí et al. [21] compared sample preparation by digestion or suspension after drying and grinding of human placenta and validated the method by use of reference material and measurements by ICP-OES. These authors observed systematic minor recoveries in the suspensions of the real samples, which were then corrected using the obtained factors. In this work, a different preparation approach was followed and fresh real samples were partly decomposed by addition of nitric acid in order to homogenize the samples. Here, validation of Fe quantification in tissues as well as in cells was achieved over a broad concentration range using GFAAS as a reference method. In addition, the results from comparison of found values in standard reference material *bovine liver* (*NIST*; *SRM 1577c*) also assure accurate quantification of Cu, Fe, Zn, and Mn. Hence, the suggested method using TXRF for detection provides multi-element quantification by fast and simple one-point internal calibration in minute sample amounts. At the same time, the straightforward and quick sample preparation allows high sample throughput.

**Table 4:** Exemplary list of methods for trace metal determination in biomedical samples. (Abbreviations: *-*, data not given; *RT*, room temperature; *GFAAS*, graphite furnace atomic absorption spectrometry; *ICP-OES*, inductively coupled plasma-optical emission spectrometry; *ICP-MS*, inductively coupled plasma-mass spectrometry; *HC*, hepatocytes; *KC*, Kupffer cells; *LSEC*, liver sinusoidal endothelial cells; *HSC*, hepatic stellate cells; *BMDMs*, bone marrow–derived macrophages)

Measurement technique	Sample type	Sample preparation (a) Reagents and treatment (b) Duration (c) Approx. sample amount	Analyzed elements	Validation by recovery in reference material or by reference method	LOD	Ref
Colorimetric assay	Rat Brain cells (astrocytes)	(a) Lysis in NaOH for 2 h, then mixing with HCl and iron-releasing reagent (mixture of HCl and KMnO_4_) for 2 h at 60 °C followed by staining with iron detection reagent (ferrozone, neocuproine, ammonium acetate, ascorbic acid) for 30 min (b) 4.5 h (c) 100 μL of cell lysates	Fe	Validated by GFAAS as reference method	-	[5]
	Mouse Liver	(a) Digestion at 90 °C in HNO_3_ and H_2_SO_4_ followed by addition of H_2_O_2_ (b) Duration not given: Incubation in ferrozine, ascorbic acid, Tris, and HCl at pH = 4 30 min (c) Not given	Fe	-	-	[4]
GFAAS	Mouse Liver Kidney Gastrocnemius muscle	(a) Microwave-assisted digestion in a mixture of HNO_3_ and H_2_O_2_; evaporated to dryness and re-dissolved in water at 200 °C (b) 40 min (c) 250 mg	Fe Cu Zn	SRM 1577c, bovine liver 95–101% 89–95% 90–95%	-	[9]
	Mouse and human Liver Macrophages	(a) Digestion in HNO_3_ followed by ultrasonication at 30–40 kHz; if necessary appropriate dilution in water (b) > 30 min (c) Not given	Fe	-	-	[7]
ICP-OES	SRM Bovine liver	(a) Microwave-assisted digestion in HNO_3_ and O_2_ (b) 35 min (c) 100/500 mg	Ca Cu Fe Mn Mg Zn	SRM 1577a, bovine liver All elements > 96%	ng/g 12 18 18 12 10 12	[11]
ICP-OES and ICP-MS	Pig Liver Pancreas Kidney Lung	(a, b) Microwave-assisted digestion using different reagent mixtures and durations: (i) HNO_3_ and H_2_O_2_ at 160 °C for 12 h (ii) HNO_3_ and H_2_SO_4_ at 160 °C for 24 h (iii) HNO_3_ at 85 °C for 12 h (iv) HNO_3_ at 175 °C for 4 h, then H_2_O_2_ at 75 °C for 2 h (c) 50 mg	Cu Zn Fe Ni	SRM 1577c, bovine liver - - - -	- - - -	[12]
ICP-MS	Human Liver Brain Kidney Bone Lung	(a) Digestion at RT in HNO_3_ for 8 h followed by 8 h at 80 °C (b) 16 h (c) 500 mg	As Be Cd Hg Mn Ni Pb Sn Tl V Cr	NRC TORT 2, lobster hepatopancreas 91% (Hg)–361% (Cr)	μg/g 0.05 0.05 0.025 0.05 0.025 0.025 0.025 0.05 0.025 0.25 0.25	[14]
	Human Liver	(a) Digestion at 120 °C for 8 h in a mixture of HNO_3_ and HClO_4_ (b) 8 h (c) Not given	Al Fe Cd Mn Cr Cu Pb Ni Zn Ag Co	IAEA-407 fish tissue -	ng/g 1.74 1.49 0.47 0.21 1.13 0.59 0.36 0.64 2.51 0.31 0.24	[10]
	Mouse Liver Spleen	(a) Digestion of homogenates at RT in HNO_3_ and H_2_O_2_ followed by drying and re-suspension in dilute HNO_3_ (b) > 12 h (c) Not given	Fe Zn Cu Mn	-	-	[15]
	Mouse Liver and spleen cells (HC, KC, LSEC, macrophages)	-	Fe	Bovine liver SRM NIST 1640 and MS1577b 86.5–88.5%	μg 0.005	[17]
ICP-MS and TXRF and GFAAS (Ni)	Human Liver	(a) Microwave-assisted digestion in HNO_3_ (b) 15 min (c) 0.5–2 mg	Cr Mn Fe Ni Cu Zn Rb Pb	SRM 1577a bovine liver ICP-MS/TXRF - 97%/119% 95%/99% - 94%/99% 102%/103% 106%/95% 93%/-	-	[13]
TXRF	Human Placenta	Drying and grinding and then (i) Digestion or … (a) Microwave-assisted digestion in HNO_3_ and H_2_O_2_ (b) 15 min (c) 500 mg … (ii) Suspension (a) Suspending in Triton X-100 solution (b) – (c) 50 mg	K Ca Fe Zn Cu As Se Sr Cd Pb	GBW08571 mussel muscle tissue (i)/(ii) 71%/64% 75%/82% 81%/84% 97%/98% 84%/143% 107%/82% 90%/88% 94%/125% 111%/91% 214%/153% And ICP-OES as reference method	μg/g (i)/(ii) 58/41 41/25 3.6/3.3 1.3/1.4 1.6/1.0 0.8/1.5 1.0/1 0.8/0.6 1.3/0.6 0.5/0.5	[21]
	Rat Kidney Spleen Liver	(a) Digestion in Parr bomb at 195 °C in HNO_3_ (b) 1 h (c) 260–300 mg	Fe Cu Zn	SRM 1577c, bovine liver -	-	[24]
	Rat Liver	(a) Microwave-assisted digestion in HNO_3_ (b) Not given (c) Not given	Ca Fe Cu Zn	-	ppm 1.29 0.195 0.101 0.109	[23]
	Mouse Liver Liver cells (KC, HC, LSEC, HSC) BMDMs	(a) Digestion at room temperature or 40 °C using HNO_3_ (b) 1–19 min (c) < 1 μg–28 mg	Fe Cu Zn Mn (Rb) (Se)	SRM 1577c, bovine liver 99 ± 10% 106 ± 8% 104 ± 1% 92 ± 11% 86 ± 10% 78 ± 9% And GFAAS as reference method	μg/L 0.32 0.46 0.44 1.06 0.38 0.32	This work

## Conclusions

This work presents the development and validation of a method for trace element quantification in minute biological samples using TXRF. Minimal sample preparation procedure was established, consisting of a partial digestion with high-purity nitric acid as the sole reagent and mixing with internal standard solution prior to application onto TXRF sample carrier. In-detail evaluation of reproducibility confirmed suitability of trace metal determination in the obtained homogenates. Diligent validation of the proposed method was successfully performed by (a) using GFAAS as a reference method for Fe determination in a large concentration range from 1 to 1600 μg/L (recovery rate, 99%) and (b) examination of the standard reference material bovine liver (SRM 1577c, NIST) for quantification of trace metals Fe, Cu, Zn, and Mn (recovery rates, 93–107%). Moreover, the obtained analytical figures of merit with detection limits in the low picogram range clearly prove suitability of the proposed method for trace metal determination in biological samples of minute amounts. In future, therefore, this method will be very valuable for investigation of biomedical samples in a valid, robust, and timesaving approach.

## Electronic supplementary material

ESM 1 (PDF 139 kb)
