# Supplementary material for: Total reflection X-ray fluorescence spectrometry for trace determination of iron and some additional elements in biological samples
Source: Anal Bioanal Chem. 2020 Apr 26;412(24):6419–29. doi: 10.1007/s00216-020-02614-8 (PMC7442763; doi:10.1007/s00216-020-02614-8)
Supplement: Supplementary file 1 — (PDF 139 kb) [file 216_2020_2614_MOESM1_ESM.pdf]

Analytical and Bioanalytical Chemistry

Electronic Supplementary Material

Total reflection X-ray fluorescence spectrometry for trace determination of iron and some additional elements in biological samples

Andreas Gruber, Riccarda Müller, Alessa Wagner, Silvia Colucci, Maja Vujić Spasić, Kerstin Leopold

Table S1 Optimized sample pretreatment parameters for TXRF and/or GFAAS measurement

	Bone marrow-derived macrophages	*Liver cells*	*Liver tissues*	
			Mouse	*Beef SRM 1577c*
**A) Sample digestion**				
Reagent	1 mL HNO_3		1 mL HNO_3	4 mL HNO_3
Digestion time and temperature	15 sec at RT	15 sec at RT	19 min at 40°C	
Sample amount	1.6 – 3.3 µg	$15{\cdot}10^3$- 1,900 $\cdot10^3$ cells	2.7 - 27.6 mg	100 mg
**B) TXRF measurement**				
1. Calibration with internal standard	10 µL of **Ga** (100 mg/L)		10 µL of **Ti** (100 mg/L)	
2. Homogenization of internal standard	1 min			
3. Applied digest volume	10 µL			
4. Drying of sample carrier	at 60 °C			
5. Measurement live time	500 s			
**C) GFAAS measurement**				
1. Calibration with external Fe standards in the concentration ranges … in µg L^{-1}	20 – 100	**HC**:20-100<br>50-160<br>70-200<br>**KC**:<br>30-200<br>**HSC**:<br>5-55<br>**LSEC**:<br>7-40<br>40-170	10-55<br>20-100<br>50-200	30 - 200
2. Sample dilution factors	2	HC:<br>11 / 14 / no dilution<br>KC/HSC/LSEC:<br>no dilution	21 / 11 / 6 / 3 / no dilution	200
3. Applied sample amount		GFAAS: 20 µL		

Table S2 Temperature program for Fe determination by GFAAS

Step	*Temperature [°C]*	*Ramp [°C s^{-1}]*	*Hold [s]*
Drying	70	4	20
Drying	90	3	20
Drying	110	5	20
Drying	125	5	20
Pyrolysis	350	50	20
Pyrolysis	950	300	10
Auto zero	950	0	6
Atomization	2300	1000	6
Cleaning	2450	500	4
